# Jet-Printable, Low-Melting-Temperature Ga–xSn Eutectic Composites: Application in All-Solid-State Batteries

**DOI:** 10.3390/ma17050995

**Published:** 2024-02-21

**Authors:** Kuan-Jen Chen, Fei-Yi Hung, Hsien-Ching Liao

**Affiliations:** 1 Department of Mechanical Engineering, Southern Taiwan University of Science and Technology, Tainan 710, Taiwan; 2 Department of Materials Science and Engineering, National Cheng Kung University, Tainan 701, Taiwan; a850425a850425@gmail.com

**Keywords:** Ga–Sn composite, natural silicate mineral, all-solid-state battery, intermetallic compounds, jet printing

## Abstract

Low-melting-point Ga–xSn eutectic composites and natural silicate mineral powders were used as the electrode and solid-state electrolyte, respectively, in all-solid-state batteries for green energy storage systems. The influences of the Sn content in the Ga–xSn composite electrode on the electrochemical performance of the batteries were evaluated, and liquid composites with a Sn concentration of up to 30 wt.% demonstrated suitability for electrode fabrication through dip coating. Sodium-enriched silicate was synthesized to serve as the solid-state electrolyte membrane because of the abundance of water molecules in its interlayer structure, enabling ion exchange. The battery capacity increased with the Sn content of the Ga–xSn anode. The formation of intermetallic compounds and oxides (CuGa_2_, Ga_2_O_3_, Cu_6_Sn_5_, and SnO_2_) resulted in a high charge–discharge capacity and stability. The Ga–Sn composite electrode for all-solid-state batteries exhibits a satisfiable capacity and stability and shows potential for jet-printed electrode applications.

## 1. Introduction

Lithium (Li)-ion secondary batteries are widely used in energy storage systems because of their high energy density and lack of memory effect. However, Li-ion batteries are costly because of the scarcity of Li mineral reserves [1]. Therefore, high-capacity and high-stability anode materials are necessary to replace Li-ion batteries. Currently, commercial Li-ion secondary batteries primarily use liquid electrolytes as a charge transmission medium and a charge carrier, with such functions critically affecting the battery performance [2]. However, these liquid electrolytes are hazardous to the environment and human health. Thus, environmentally friendly solid-state electrolytes have been developed as liquid electrolyte substitutes and introduced into all-solid-state energy storage systems [3,4].

Studies on solid-state electrolyte materials have employed lithium superionic conductor (LISICON)-like [5,6,7], perovskite [8], Na super ionic conductor (NASICON)-like [9], and garnet [10] materials. However, such solid-state electrolytes are potentially difficult to use in practical applications. The ionic conductivity at the grain boundaries of perovskite structures is too low (<10^−5^ Scm^−1^) at room temperature. Sodium fast ion conductors exhibit poor compatibility with electrodes, and the surface of garnet is easily oxidized to form a passivation layer, which increases interfacial impedance. Silicate minerals are a clay mineral mainly composed of montmorillonite, which is formed from a central octahedral alumina sheet between two tetrahedral silica sheets [11]. The montmorillonite structure contains a considerable amount of interlayer water that contains cations; thus, it can maintain the charge balance. Notably, natural silicate minerals are rarely used as solid-state electrolytes for ion batteries. In numerous studies of all-solid-state batteries, Li-based composites and Li-containing metals have been used for electrolyte and electrode materials [12,13,14]. However, the scarcity of Li mineral reserves and battery recycling are inevitable problems in the future. Therefore, this study examined Na-based silicate minerals that are both nontoxic and abundant in the earth’s crust and therefore suitable for use as an electrolyte in secondary ion batteries.

Gallium (Ga) is a low-melting-point metal (30 °C) and one of the few metals that exhibits a liquid phase at room temperature. Ga metal and Ga compounds are both nontoxic, meeting the requirements for environmental friendliness as an electrode material in energy storage products. Furthermore, Ga metal possesses an inhibiting phase transition and self-healing properties, both of which promote the cycling stability of batteries [15,16,17]. In addition, LiGaO_2_ compounds are produced by the reaction of Ga and Li ions, which could reduce the lattice transformation during the insertion and extraction of ions [18]. Although Ga electrodes for batteries exhibit considerable cycling stability, their low theoretical capacity is a concern, but can be improved. The price (USD/kg) of tin (Sn) metal is about 10% of that of Ga metal, and it has a considerable theoretical specific capacity (993.4 mAh/g) [19]. Adding Sn to Ga can reduce the cost and improve the capacity of Ga-based anode materials [20]. Sn metal exhibits severe volume expansion during charge–discharge processes, thereby degrading the battery performance [21]. However, the combination of Sn and Ga can reduce the volume expansion of Sn during alloying/dealloying processes; thus, Ga–Sn composites were chosen as an anode material in this study. In addition, low melting-point Ga–xSn composites can be jet-printed to fabricate thin-film electrodes for ion batteries in the future.

This study used Ga–xSn composite anodes, a solid-state Na silicate electrolyte (NSE; chemical composition: SiO_2_: 63.02 wt.%, Al_2_O_3_: 21.08 wt.%, Fe_2_O_3_, FeO: 3.25 wt.%, MgO: 2.67 wt.%, Na_2_O, K_2_O: 2.57 wt.%, CaO: 0.65 wt.%) [22], and a Sn foil cathode for the assembly of all-solid-state batteries. To increase the Na content in the NSE, Na silicate mineral powder was mixed with saturated aqueous sodium phosphate at a ratio of 2 g:3 mL. The contribution of the solid-state electrolyte’s ionic transport properties to the performance of the batteries was examined. Ga–xSn anode materials with various Sn concentrations were employed to evaluate their effects on the charge–discharge characteristics of all-solid-state batteries.

## 2. Materials and Methods

**Figure 1:**
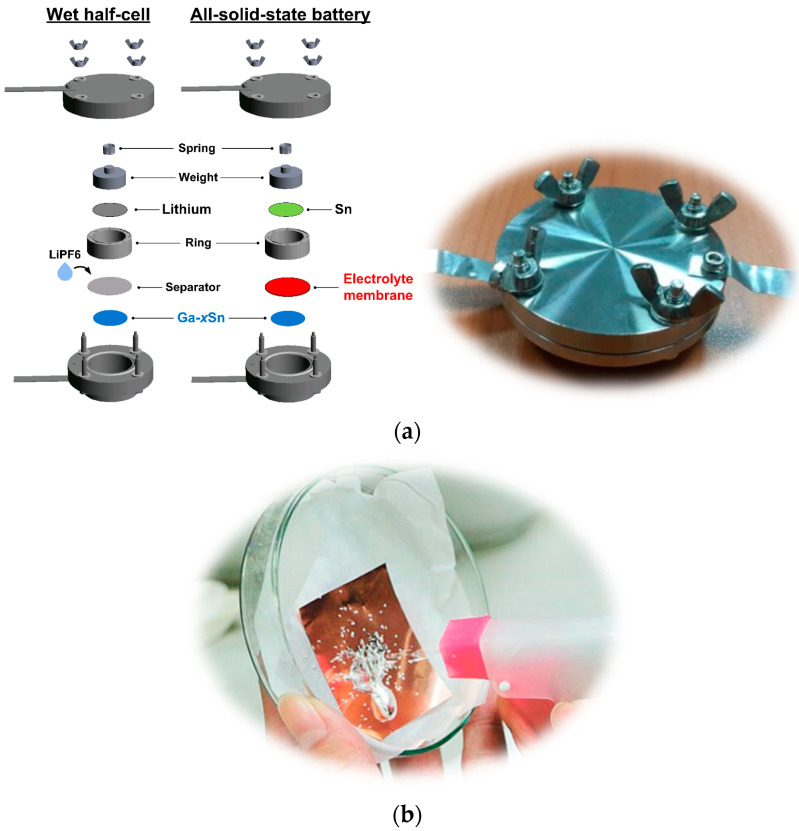
(**a**) Internal structure and photograph of battery testing cells. (**b**) Jet-print testing of liquid Ga–xSn composite.

To evaluate the influence of adding Sn to the Ga anode of batteries on the charge–discharge characteristics, a Li metal (counter electrode) and lithium hexafluorophosphate (LiPF_6_) electrolyte were used for wet half-cell performance testing [23]. Because the Ga–xSn eutectic composite was superior in performance to the pure Ga electrode in wet-cell testing [24], the Ga–xSn electrodes were later used for testing in the dry-cell battery (Figure 1a). The melting point and viscosity of Ga–xSn composite increased with an increase in the Sn concentration [25]; thus, the proportion of the present composite was adjusted to meet the requirements of the coating process. Testing of the molten Ga–xSn composite coated on Cu foil with a manual water sprayer was performed (Figure 1b), indicating that the molten Ga–xSn has feasibility and potential in the jet-printing process.

Pure liquid Ga (Purity: 99.99%, Victors Enterprise Co., Ltd., Tainan, Taiwan) and pure solid Sn metal (Purity: 99.99%, JAC) were mixed at 9:1, 8:2, and 7:3 weight ratios and then heated to 250 °C to form uniform Ga–xSn composite electrodes; the Ga–xSn composites were denominated as Ga–10Sn, Ga–20Sn, and Ga–30Sn, respectively, according to the weight of Sn used. The molten Ga–xSn composites (120 °C) were dip-coated onto pure copper (Cu 99.9%, Thermo Fisher Scientific, Walthman, MA, USA) foil. Each of the coated Ga–xSn foils was subjected to heat treatment (200 °C for 30 min) in an air furnace to ensure the interface between the Ga–xSn and Cu foil was closely bonded. The Ga–xSn foils were then cut into disks (14 mm in diameter and approximately 200 μm thick) to be used as anodes. A rolled Sn foil (400 μm thick) was used as a counter electrode (cathode). Subsequently, 2 g of silicate mineral powder (Montmorillonite 99%, American Colloid Company, Hoffman Estates, IL, USA) and 3 mL of a saturated sodium phosphate (99%, Nihon Shiyaku, Kyoto, Japan) aqueous solution were mixed to be used as a solid-state NSE. In order to improve the adhesion between the electrolyte and the electrode, the NSE was hot-pressed onto a polymer membrane and used in the all-solid-state batteries (Ga–xSn/NSE/Sn) fabricated in the present study (Figure 1).

To evaluate the electrochemical reactions of the wet half-cell batteries, cyclic voltammetry was performed with an advanced potentiostat (PARSTAT 2273, Princeton Applied Research, Princeton, NJ, USA) at room temperature (25 °C). The electric potential was limited to the range of 0.0–2.0 V, and the scanning speed was fixed at 2 mVs^−1^. Charge–discharge testing in wet half-cell and all-solid-state batteries were performed using a battery testing cell and an automatic battery tester (BAT-750B, Acutech Systems Co., Ltd., Taichung City, Taiwan). Under a constant temperature of 25 °C, the cells were charged at a constant voltage of 5 V for 10 min and discharged at a constant current (0.01 mA). The cut-off voltage was 5 mV. The phase composition of the anode electrode was examined via X-ray diffractometry (XRD; D8 Discover, Bruker, Madison, WI, USA) with Cu-Kα radiation. The surface morphologies of the electrodes were examined via scanning electron microscopy (SEM; SU-5000, Hitachi, Japan). Furthermore, the phase composition of the NSE was identified through Fourier- transform infrared (FTIR, Spectrum Two, PerkinElmer Inc., Walthman, MA, USA) spectroscopy in transmission mode with a frequency range of 500–4000 cm^−1^ and a resolution of 1 cm^−1^s^−1^. After charge–discharge testing, the interfacial characteristics of the electrodes were determined through focused ion beam (FIB; Helios G3CX, FEI, USA) analysis with energy-dispersive microscopy (EDS) to understand the status of ion migration.

## 3. Results

### 3.1. Characteristics of Ga–xSn Electrodes for a Wet Half-Cell

**Figure 2:**
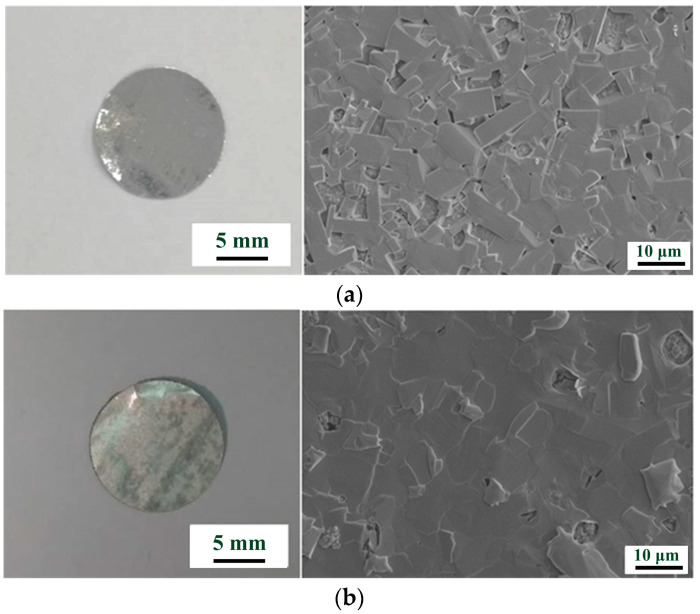
Macrograph and surface morphology of spray-coated (**a**) Ga and (**b**) Ga–10Sn anodes.

Figure 2 presents images of the coated Ga and Ga–10Sn anodes. The surface of the Ga–10Sn electrode was smoother and denser than that of the pure Ga electrode. This result indicates that Ga–Sn composites are more viscous than molten pure Ga; thus, adding Sn increases agglomeration during solidification [26,27]. The solid-phase protrusions on the surface of the Ga–10Sn electrode resulted from Sn segregation [28]. The solidification rate of the composite was inconsistent, which caused some holes to form on the surface, but this can be improved by heat treatment.

**Figure 3:**
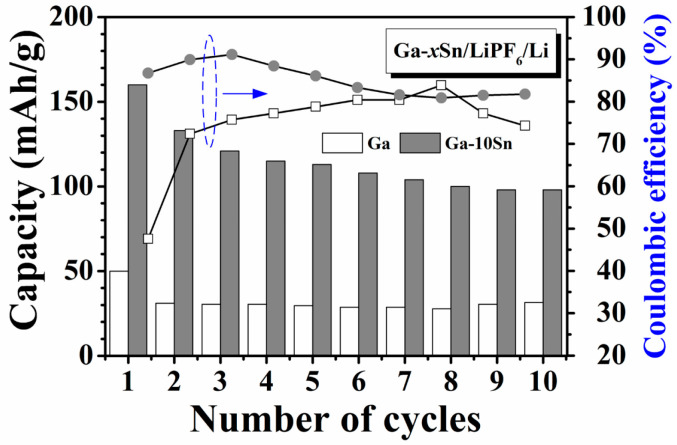
Cycling performance of wet half-cells using Ga and Ga–10Sn electrodes.

Figure 3 displays the cycling performance obtained through the charge–discharge testing of the half-cells using the Ga and Ga–10Sn electrodes. The capacities of both half-cells were the highest in the first cycle but decreased and stabilized in subsequent cycles. This result indicates the formation of a solid–electrolyte interface (SEI) layer in the first cycle [23]. Notably, the capacity decays in Ga–10Sn, which is associated with the growth rate of the SEI layer. With the results of the initial charge–discharge cycle excluded, the average capacity of the pure Ga electrode was 32 mAhg^−1^ with a mass loading of 700 mg/cm^2^, and the pure Ga electrode exhibited excellent cycling stability (Cyclic stability (%) = $\frac{Capacity\;in\;the\;last\;cycle}{Highest\;capacity}\times 100\%$) (Table 1) [18]. However, the average capacity of the Ga–10Sn electrode was greater at 111 mAhg^−1^, but the cycling stability was only 75% of that of the pure Ga electrode. The Ga–10Sn electrode exhibits acceptable cycling stability but the capacity retention rate gradually improves in subsequent cycles. Additionally, adding Sn to Ga can reduce the cost of electrode materials. Furthermore, the Ga–10Sn electrode exhibits a considerable initial coulombic efficiency (86.8%), higher than that of the pure Ga electrode (47.6%). This result reveals that adding Sn to Ga can enhance the reversibility of the electrode material, which increases the number of cation carriers. Therefore, the Ga–10Sn electrode achieved a better charge–discharge performance than the pure Ga electrode.

**Figure 4:**
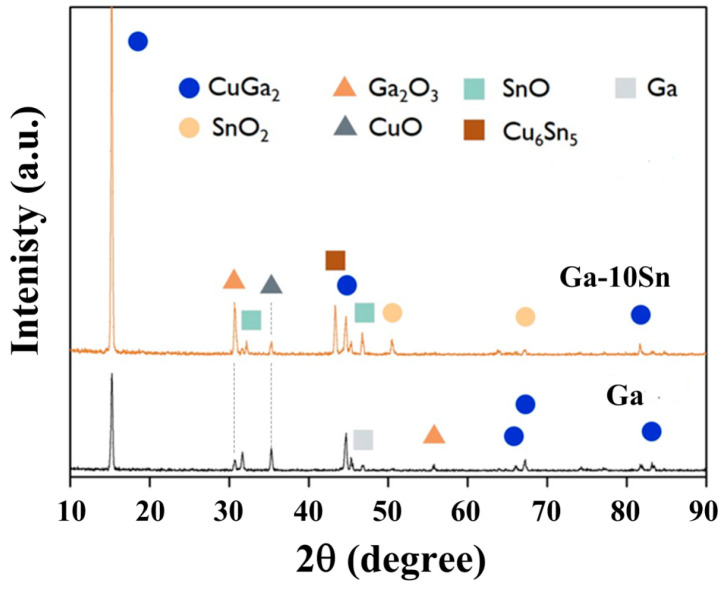
X-ray diffraction patterns of Ga and Ga–10Sn electrodes obtained after heat treatment of 200 °C.

To assess the effects of the electrode phase composition on battery performance, both electrodes were examined via XRD. Figure 4 displays the XRD spectra of the pure Ga and Ga–10Sn electrodes, which were mainly composed of Ga, Ga_2_O_3_, and CuGa_2_ phases (JCPDS Card No. 04-0673 and 41-11-3) [29]. Ga_2_O_3_ and CuGa_2_ phases existed in both electrodes, indicating that Ga combined with O and thermally diffused Cu atoms (from the Cu substrate) during atmospheric heat treatment (200 °C for 30 min). Notably, SnO and Cu_6_Sn_5_ phases were present in only the Ga–10Sn electrode. CuGa_2_ can be decomposed into Ga and Cu during the insertion and extraction of ions, thereby enhancing a battery’s cycling stability [30], and Ga_2_O_3_ is reduced to Ga0 to repair the electrode material, helping to maintain cycling stability [17]. Moreover, SnO_2_ can act as the active center of Li ion adsorption during the insertion and extraction of ions, thereby improving the battery capacity [31]. Additionally, Cu_6_Sn_5_ can be decomposed into Sn and Cu during the charge–discharge process to increase both the battery’s capacity and stability [32]. In summary, the Ga_2_O_3_ and CuGa_2_ phases help to maintain cycling stability, and the formation of the SnO and Cu_6_Sn_5_ compounds provides additional vacancies during the insertion and extraction processes, thus resulting in the high capacity of the Ga–10Sn electrode.

**Figure 5:**
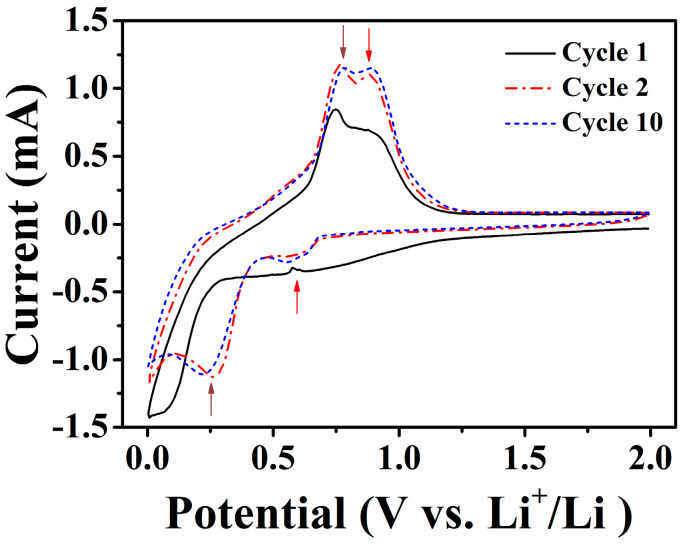
Cyclic voltammograms of a Ga–10Sn/LiPF_6_/Li battery.

The electrochemical reaction between the Ga–10Sn electrode and Li ions was evaluated via cyclic voltammetry (Figure 5). An irreversible reaction occurred between 1.2 and 0.7 V during the first cycle, and two prominent peaks appeared during the oxidation reaction after the first cycle, which are attributed to the formation of the SEI layer. The oxidation potentials of 0.75 and 0.8 V correspond to Li^+^ ions being extracted from the Ga and Sn sites in the Ga–xSn electrode, respectively [20,33]. The peaks at 0.3 and 0.61 V during the reduction reaction indicate the alloying of Li^+^ ions into the Ga and Sn ions, respectively, in the Ga–xSn electrode. This result indicates that the superior capacity performance of the Ga–10Sn electrode is due to the increase in oxidation and reduction potentials caused by the Sn and Li ions. Based on the above results, the Sn-doped Ga electrode indeed promotes the battery’s performance. Therefore, the Ga–xSn electrode was assembled into a solid-state battery and the battery performance was evaluated.

### 3.2. Assembly and Testing of the Ga–xSn Electrode in All-Solid-State Batteries

**Figure 6:**
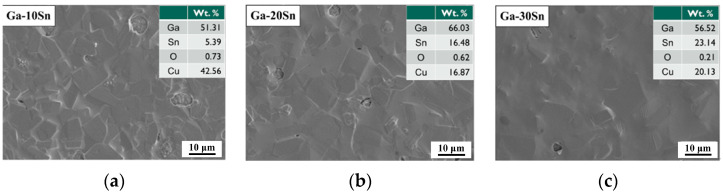
Surface morphologies of Ga–xSn anodes after 200 °C heat treatment: (**a**) Ga–10Sn, (**b**) Ga–20Sn, and (**c**) Ga–30Sn.

Because the Ga–10Sn electrode exhibited a superior performance compared to the pure Ga electrode, Ga–Sn composite electrodes with various Sn concentrations were further investigated for use in an all-solid-state battery (Ga–xSn/NSE/Sn). Figure 6 presents the surface morphologies of the Ga–10Sn, Ga–20Sn, and Ga–30Sn anodes. There is less segregation on the electrode surface is at higher temperature (e.g., 120 °C) because of the higher diffusion rate, resulting in a more uniform distribution in the composite coating. The viscosity of molten Ga–xSn composites increased with the Sn concentration [26]. Thus, the Ga–30Sn electrode surface was the most uniform because the particles of the high-viscosity molten composite readily agglomerated and were easily interdiffused. An EDS analysis indicated that the weight ratio of Ga to Sn in the composite electrodes was as specified by the experimental design.

**Figure 7:**
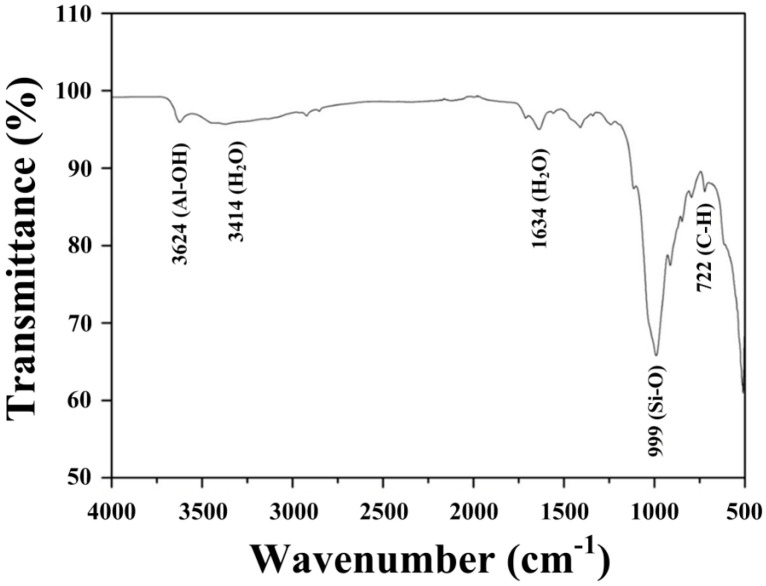
Fourier transform infrared spectroscopy analysis of the synthesized solid−state electrode.

To evaluate the NSE membrane composition, the synthesized electrolyte was examined via EDS analysis (Figure 7). The NSE was mainly composed of silicate (999 cm^−1^) and aluminate (3624 cm^−1^) compounds [34,35]. The bands at 3414 cm^−1^ and 1634 cm^−1^ correspond to the hydrogen bonding of H_2_O [36]. This result indicates that the interlayer in the solid electrolyte structure contains abundant water molecules, which promote ion exchange.

**Figure 8:**
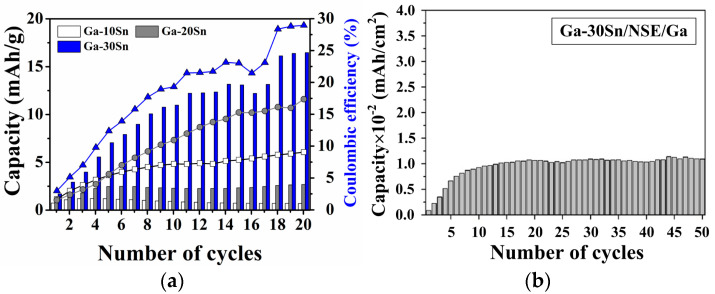
Cycling performances of (**a**) Ga–xSn/NSE/Sn and (**b**) Ga–xSn/NSE/Ga batteries.

Figure 8a shows the cycling performance of the all-solid-state battery using Ga–xSn anodes with different Sn concentrations (x = 10, 20, and 30 wt.%). Unlike the liquid electrolyte, an SEI was not formed on the solid electrolyte’s surface; thus, the all-solid-state battery did not exhibit a significant decrease in capacity after the first cycle. The average capacity of the battery increased with the Sn concentration. The Sn concentration used in the Ga–xSn composites in this study did not exceed 30 wt.% because the viscosity of the molten composite increases with the Sn concentration; higher viscosities at higher Sn concentrations may result in difficulties in jet printing applications. In addition, the Ga–30Sn and Na silicate electrolytes and Ga metal were used for the assembly of all-solid-state batteries. In this case, the dry-cell battery maintained stable charge–discharge characteristics (Figure 8b). In the dry-cell battery system, cations are transported through lattice defects in the solid-state electrolyte [37,38]. Based on the above result, the Ga ions were the primary cation carriers in the all-solid-state battery, while Na ions in the solid electrolyte also served as charge carriers to promote ion transmission. Therefore, the contribution of Ga ions in the battery is confirmed via the cycling performance of the all-solid-state battery.

**Figure 9:**
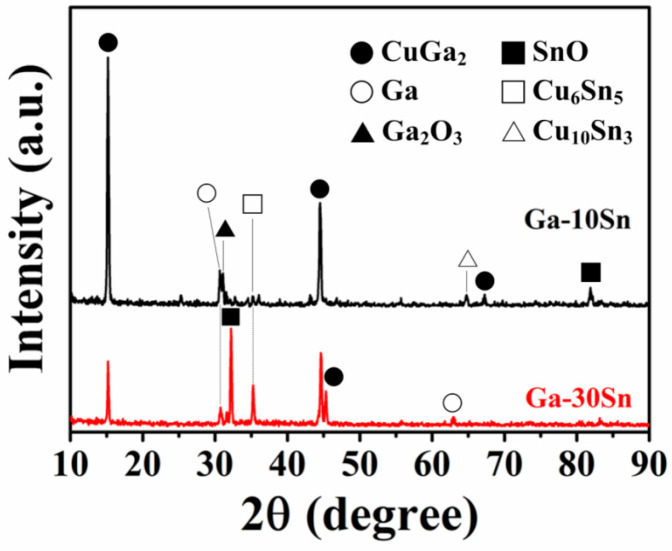
X-ray diffraction patterns of Ga–10Sn and Ga–30Sn electrodes.

Figure 9 displays the XRD patterns of the Ga–10Sn and Ga–30Sn electrodes after heat treatment at 200 °C. The anodes were composed of Ga, CuGa_2_, Ga_2_O_3_, SnO, and Cu6_S_n_5_ phases, which confirmed that thermally diffused Cu atoms had reacted with the Ga–30Sn composite. The peak diffraction intensities for the SnO and Cu_6_Sn_5_ phases increased as the Sn concentration was increased from 10 to 30 wt.%. This result indicates that a higher concentration of Sn in the Ga–30Sn anode enabled the formation of more Sn compounds, promoting the cycling stability [39] and improving the capacity [40,41].

**Figure 10:**
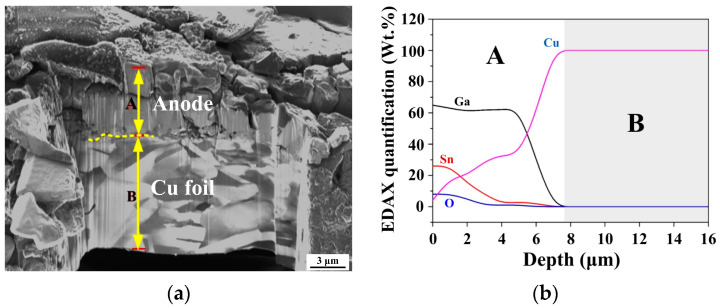

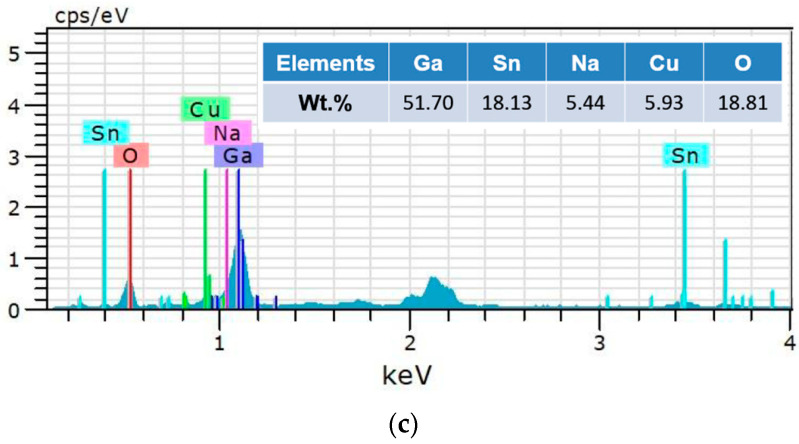
(**a**) Cross-sectional image and (**b**) depth profile of Ga–30Sn anode obtained after 200 °C heat treatment. (**c**) EDS analysis of Ga–30Sn electrode surface obtained after charge–discharge testing.

Figure 10a presents a cross-sectional image of the Ga–30Sn electrode after heat treatment. The average thickness of the Ga–30Sn electrode was approximately 6 µm. The depth profile in Figure 10b reveals decreases in the Ga and Sn concentrations toward the Cu matrix and that Cu atoms diffused to the surface layer (diffusion depth: ~7 μm). This result indicates that the interface between the Ga–30Sn composite and Cu foil is firmly bonded after heat treatment. Furthermore, an oxygen signal appeared after heat treatment, attributed to the formation of SnO. The formation of these compounds on the electrode surface is consistent with the results of the XRD analysis (Figure 9). After the charge–discharge cycling test, some Na ions were observed on the electrode surface (Figure 10c). This result indicates that Na ions can pass through the interface between the electrode and the NSE electrolyte, which can act as a charge carrier during the insertion and extraction process.

According to the aforementioned results, the Ga–30Sn electrode and the NSE have potential as an anode and an electrolyte for all-solid-state batteries, respectively. Ga and Na ions act as charge carriers in the all-solid-state battery, which improves the battery’s performance. In the future, the Ga–30Sn anode and cathode materials can be optimized for the all-solid-state battery structure. The effects of operating temperature and current charge on the performance of such all-solid-state batteries also require additional study.

## 4. Conclusions

This study evaluated the cycling performance of pure Ga and Ga–xSn composite electrodes in a wet half-cell battery. The addition of Sn to the Ga electrodes improved the battery’s capacity while maintaining a considerable charge–discharge cycling stability, indicating the higher reversibility of the electrode material. Subsequently, the Ga–xSn composite electrode, a synthesized Na silicate electrolyte membrane, and a rolled Sn foil cathode were incorporated into all-solid-state batteries for charge–discharge cycling. FTIR analyses revealed an abundance of interlayer water molecules inside the synthesized NSE structure, which promoted ion exchange. Ga–xSn composites with a Sn concentration of up to 30 wt.% (Ga–30Sn) were demonstrated to be suitable for dip coating, and the battery capacity increased with the Sn concentration in the Ga–xSn electrode. The Ga–30Sn anode was composed of CuGa_2_, Ga_2_O_3_, Cu_6_Sn_5_, and SnO_2_ phases, which promoted the capacity and cycling stability of the battery. In sum, both the Ga and Na ions in the solid-state battery act as charge carriers, which contributes to the improvement in the charge–discharge performance of battery. In addition to electrode materials, improving the ionic conductivity of solid-state electrolytes is one of the key factors to promote all-solid-state battery performance [42]. The characteristics of solid electrolytes and electrode material coating technology still need to be improved. The low melting temperature of Ga–xSn composites will be helpful for the development of jet-printed electrodes. All-solid-state Ga–xSn batteries with Ga–xSn electrodes have stable and appropriate charge–discharge characteristics, making them suitable for flexible all-solid-state energy storage applications.

## Figures and Tables

**Table 1:** Cycling performances of Ga and Ga–10Sn electrodes.

Electrode	Average Capacity (mAh/g)	Cyclic Stability (%)
Ga	32	100
Ga–10Sn	111	75

## Data Availability

Data are contained within the article.
